# Supplementary material for: Prominin-1 Regulates Retinal Pigment Epithelium Homeostasis: Transcriptomic Insights into Degenerative Mechanisms
Source: Int J Mol Sci. 2025 Nov 28;26(23):11539. doi: 10.3390/ijms262311539 (PMC12692251; doi:10.3390/ijms262311539)
Supplement: Supplementary file 1 [file ijms-26-11539-s001.zip › ijms-3983807-supplementary/Supplementary Files/Supplemental Files with figure legends.pdf]

Fig. S1

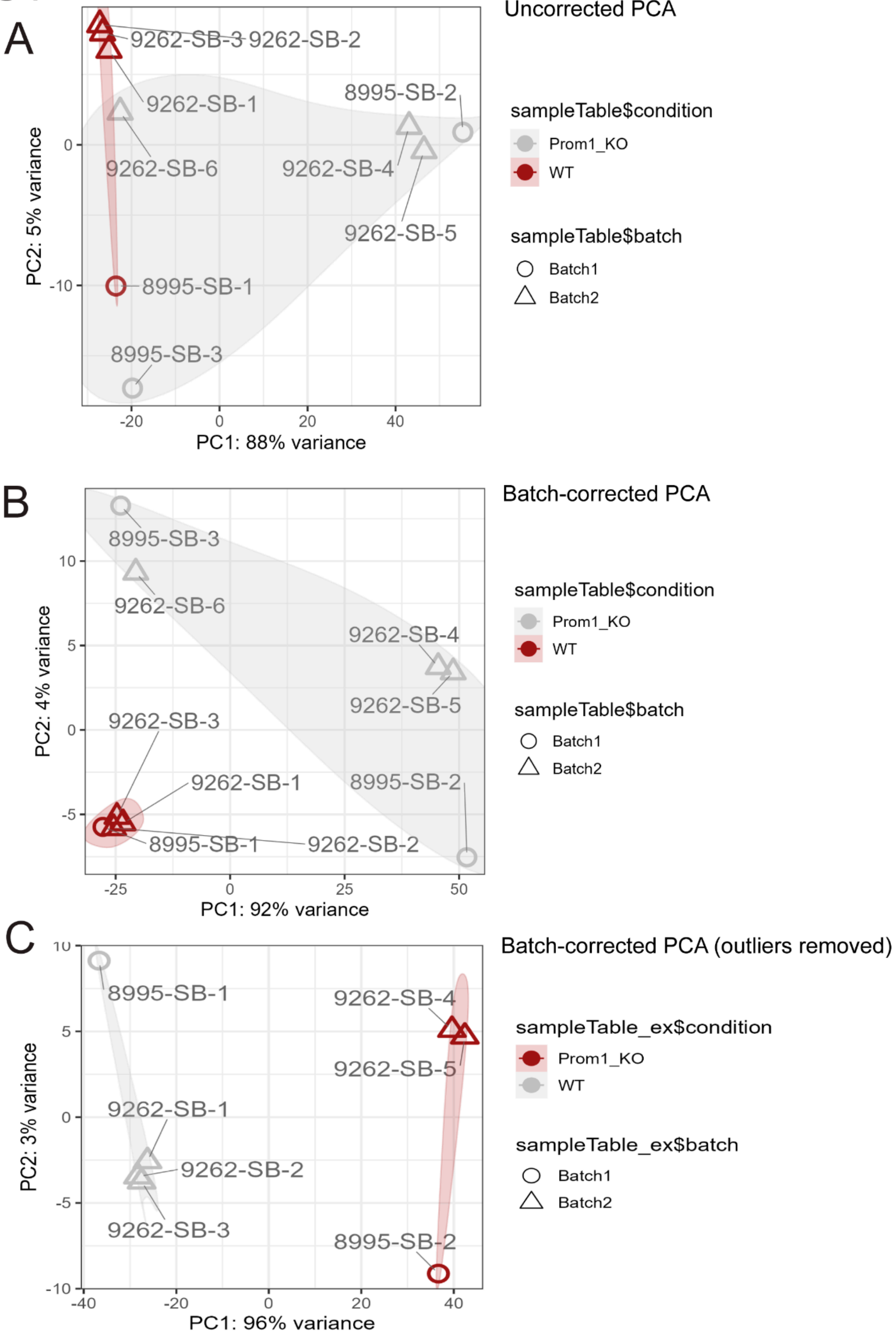
A
Uncorrected PCA
9262-SB-3
9262-SB-2
9262-SB-1
9262-SB-6
8995-SB-2
9262-SB-4
9262-SB-5
8995-SB-1
8995-SB-3
PC2: 5% variance
PC1: 88% variance
sampleTable$condition
Prom1_KO
WT
sampleTable$batch
Batch1
Batch2
B
Batch-corrected PCA
8995-SB-3
9262-SB-6
9262-SB-4
9262-SB-5
9262-SB-3
9262-SB-1
8995-SB-2
8995-SB-1
9262-SB-2
PC2: 4% variance
PC1: 92% variance
sampleTable$condition
Prom1_KO
WT
sampleTable$batch
Batch1
Batch2
C
Batch-corrected PCA (outliers removed)
8995-SB-1
9262-SB-4
9262-SB-5
9262-SB-1
9262-SB-2
9262-SB-3
8995-SB-2
PC2: 3% variance
PC1: 96% variance
sampleTable_ex$condition
Prom1_KO
WT
sampleTable_ex$batch
Batch1
Batch2

Fig. S1. Sample-level quality control by principal component analysis (PCA) of WT and Prom1-KO mRPE transcriptomes. (A) A dot plot with each sample shown with PC1 on the x-axis and PC2 on the y-axis. Uncorrected PCA shows that PC1 accounts for the majority of the variance (88%), while PC2 accounts for only 5%. (B) Batch-corrected PCA shows that PC1 accounts for 92% of the variance and PC2 for 4%. (C) Batch-corrected PCA, after removing outliers, shows good separation between conditions, with 96% of the variance in PC1.

Fig. S2

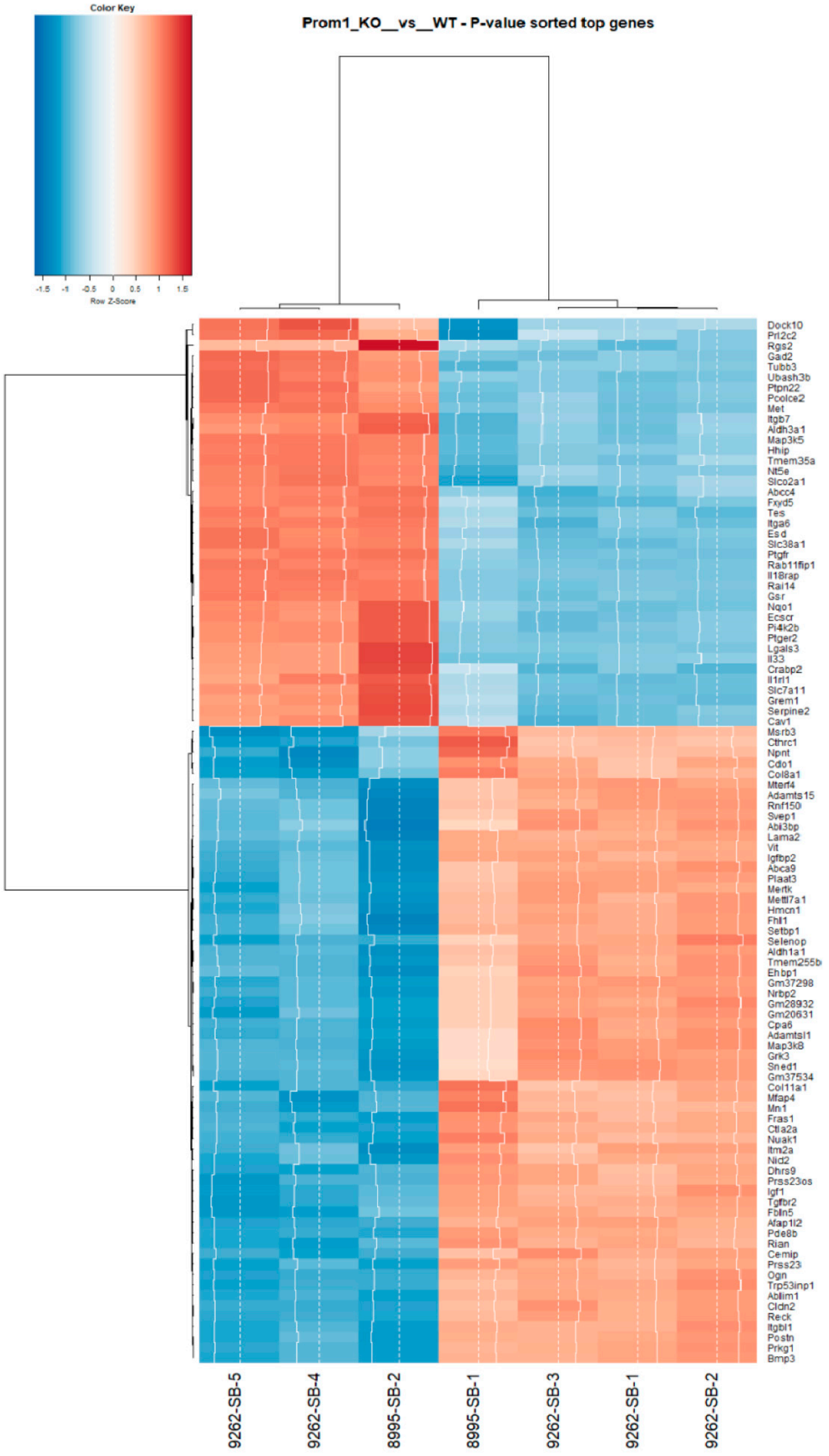

Color Key
-1.5 -1 -0.5 0 0.5 1 1.5
Row Z-Score
Prom1_KO__vs__WT - P-value sorted top genes
Dock10
Prl2c2
Rgs2
Gad2
Tubb3
Ubash3b
Ptpn22
Pcolce2
Met
Itgb7
Aldh3a1
Map3k5
Hhip
Tmem35a
Nt5e
Slco2a1
Abcc4
Fxyd5
Tes
Itga6
Esd
Slc38a1
Ptgfr
Rab11fip1
Il18rap
Rai14
Gsr
Nqo1
Ecscr
Pi4k2b
Ptger2
Lgals3
Il33
Crabp2
Il1rl1
Slc7a11
Grem1
Serpine2
Cav1
Msrb3
Cthrc1
Npnt
Cdo1
Col8a1
Mterf4
Adamts15
Rnf150
Svep1
Abi3bp
Lama2
Vit
Igfbp2
Abca9
Plaat3
Mertk
Mettl7a1
Hmcn1
Fhl1
Setbp1
Selenop
Aldh1a1
Tmem255b
Ehbp1
Gm37298
Nrbp2
Gm28932
Gm20631
Cpa6
Adamtsl1
Map3k8
Grk3
Sned1
Gm37534
Col11a1
Mfap4
Mn1
Fras1
Ctla2a
Nuak1
Itm2a
Nid2
Dhrs9
Prss23os
Igf1
Tgfbr2
Fbln5
Afap1l2
Pde8b
Rian
Cemip
Prss23
Ogn
Trp53inp1
Ablim1
Cldn2
Reck
Itgbl1
Postn
Prkg1
Bmp3
9262-SB-5
9262-SB-4
8995-SB-2
8995-SB-1
9262-SB-3
9262-SB-1
9262-SB-2

Fig. S2. Heatmap of top 100 differentially expressed genes between Prom1-KO and WT groups. Hierarchical clustering heatmap shows the top 100 DEGs ranked by P-value. Color intensity indicates relative expression levels, with red representing upregulation and blue representing downregulation. The clustering of samples is based on similarity in expression profiles. The color key (top left) shows the scale of normalized expression values.

Fig. S3

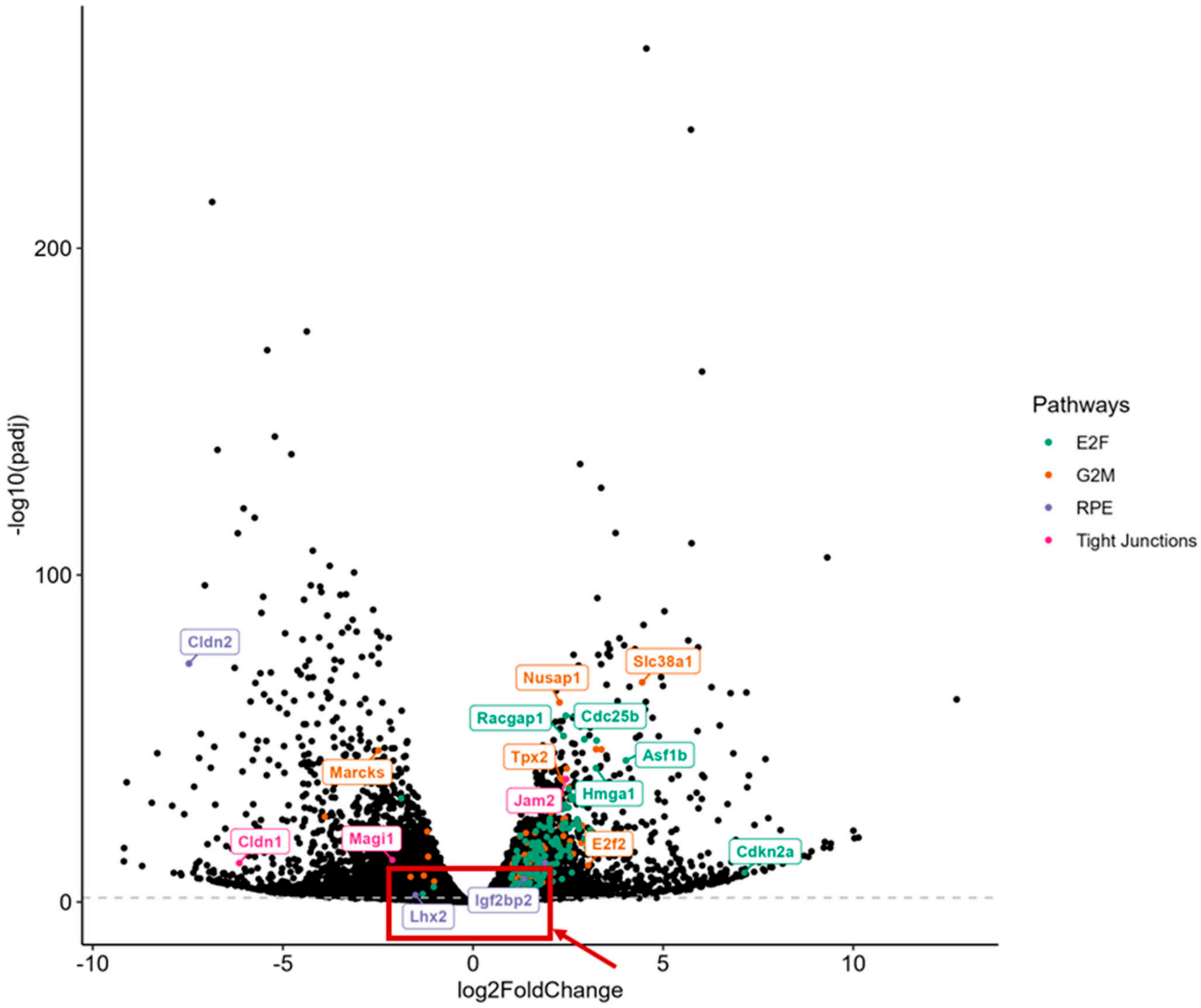

Fig. S3. Loss of Prom1 regulates tight junction and proliferation pathway genes in mouse RPE cells. A volcano plot of RNA-sequencing data highlighting the differentially expressed genes associated with the

E2F, G2M, RPE, and tight junctions' pathways using stringent differential expression criteria (Log2FC>1, adjusted p-value >=0.05). Genes significantly downregulated include Cldn2, Cldn1, and Lhx2, indicating defects in the outer blood-retinal barrier and downregulation of the RPE differentiation program, leading to a less functional RPE. Upregulated genes include E2F2, Igf2bp2, Hmga1, and Cdkn2a, suggesting a shift from a pro-senescent RPE phenotype to one that promotes proliferation, survival, and a potential loss of RPE function.
