# Supplementary figures and images for: Prominin-1 Regulates Retinal Pigment Epithelium Homeostasis: Transcriptomic Insights into Degenerative Mechanisms
Source: Int J Mol Sci. 2025 Nov 28;26(23):11539. doi: 10.3390/ijms262311539 (PMC12692251; doi:10.3390/ijms262311539)

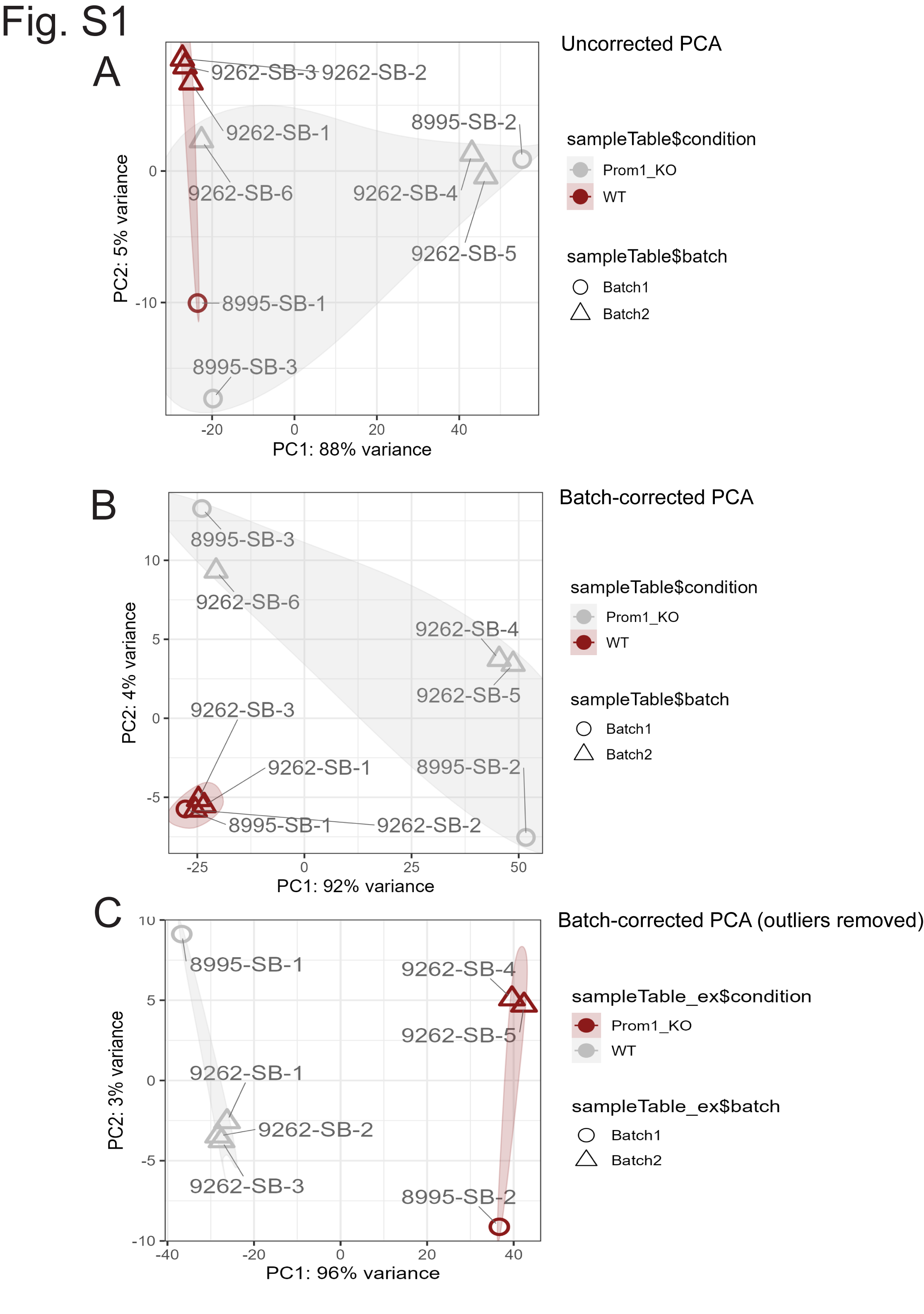

Supplement: Supplementary file 1 [file ijms-26-11539-s001.zip › ijms-3983807-supplementary/Supplementary Files/Fig. S1.tif]

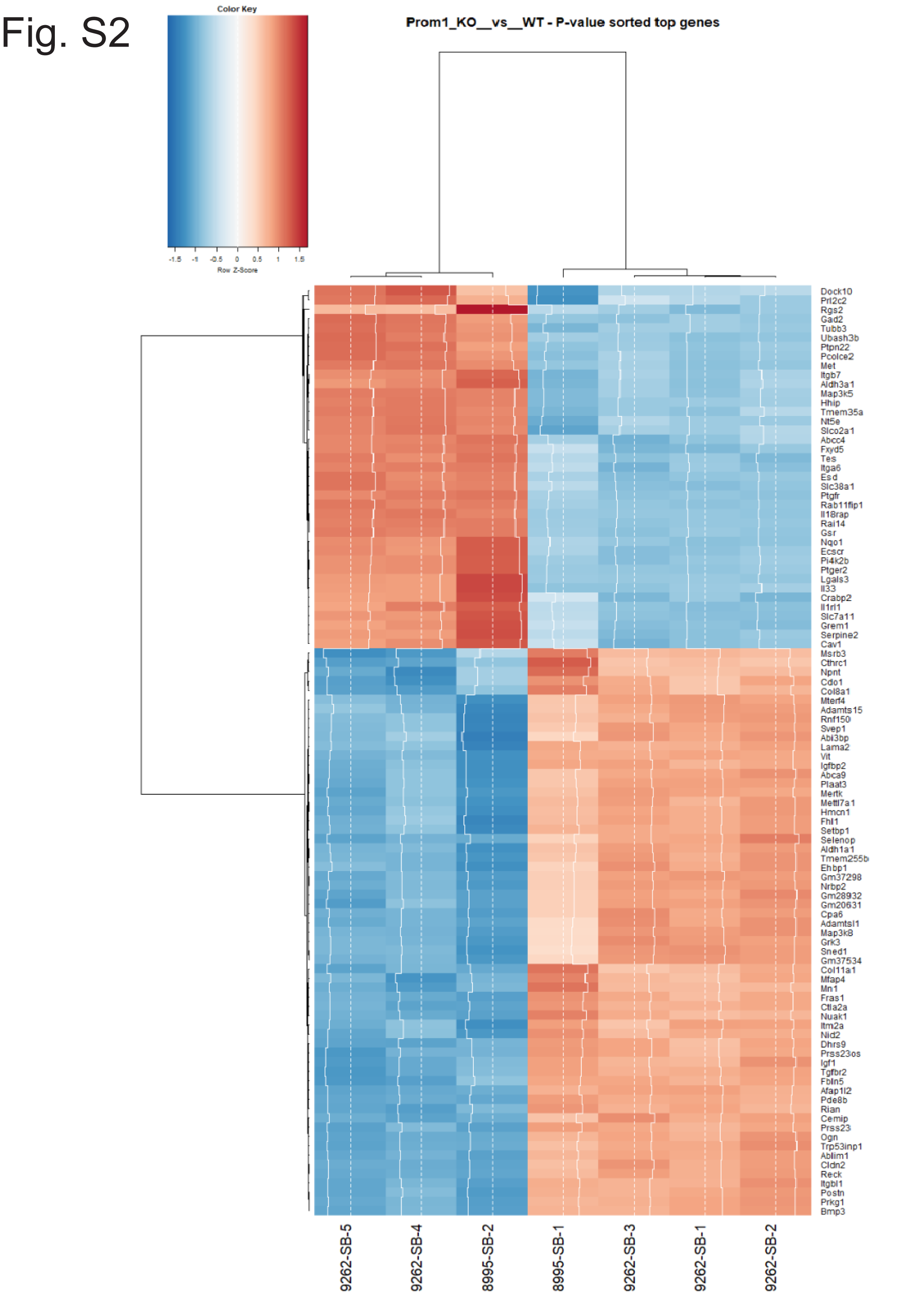

Supplement: Supplementary file 1 [file ijms-26-11539-s001.zip › ijms-3983807-supplementary/Supplementary Files/Fig. S2.tif]

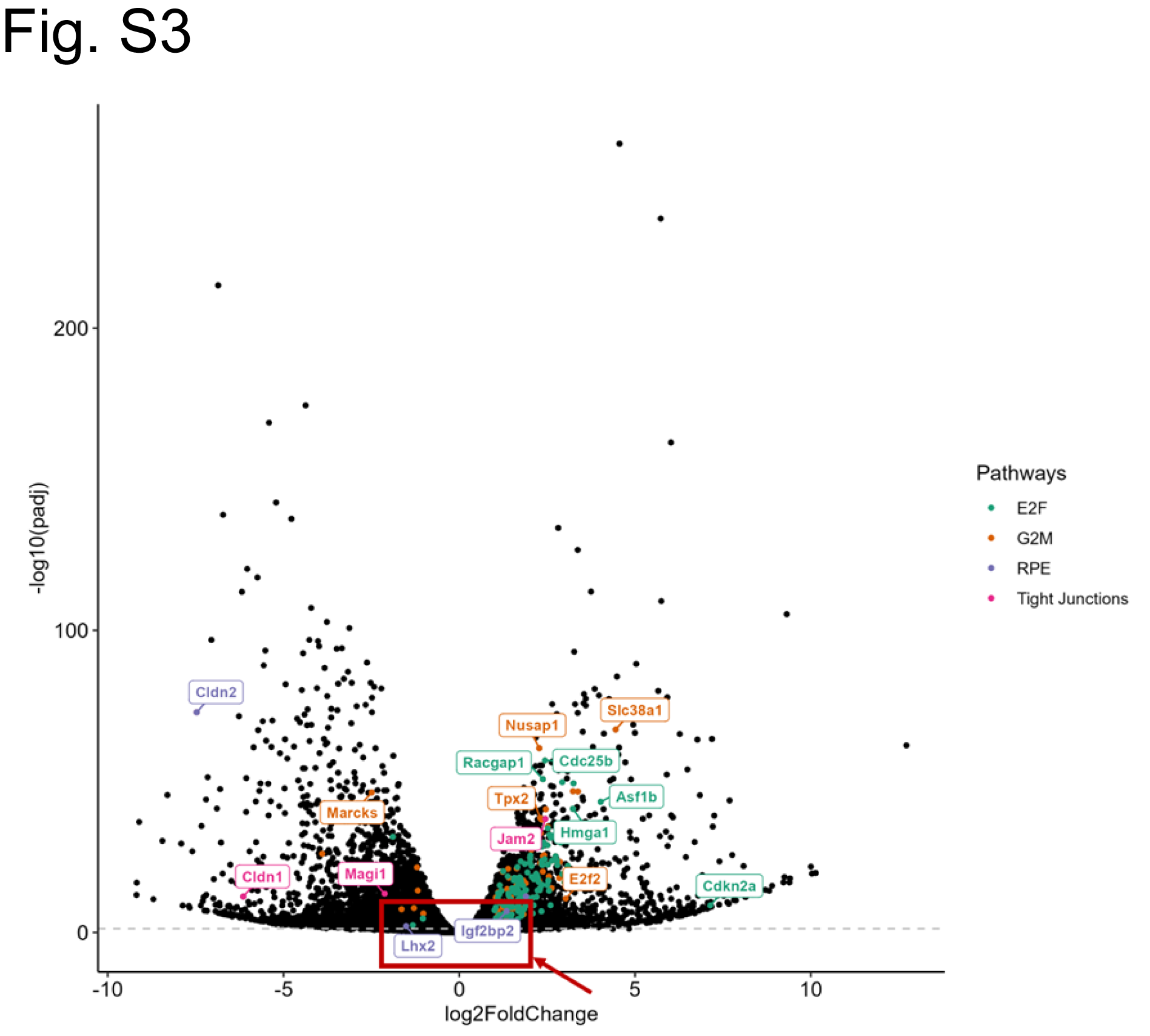

Supplement: Supplementary file 1 [file ijms-26-11539-s001.zip › ijms-3983807-supplementary/Supplementary Files/Fig. S3.tif]
